# Society Meetings

**Published:** 1907-03

**Authors:** 


### SOCIETY MEETINGS.

The Buffalo Academy of Medicine held meetings during the months of February and March as follows:

*Section on Surgery.*—Tuesday evening, February 5. Program: Treatment of Rodent Ulcer on the Face, James Bell, Attending Surgeon Royal Victoria Hospital, Montreal, Canada.

*Section on Medicine.*—Tuesday evening, February 12. Program: (a) Diseases of the naso-pharynx in infancy, John Lovett Morse, Associate Professor of Pediatrics, Harvard Medical School, Boston; Discussion opened by Dr. Irving M. Snow; (b) Some effects of spirit and drug taking on the upper air passages, T. D. Crothers, Superintendent of Walnut Lodge Hospital, Hartford, Conn.

*Section on Pathology.*—Tuesday evening, February 19. Program: Spirochetes, illustrated with lantern slides, F. G. Novy, University of Michigan, Ann Arbor, Mich.

*Section on Obstetrics and Gynecology.*—Tuesday evening, February 26. Program: (a) Presentation and report of a case of melena neonatorum following cesarian section, Wm. G. Taylor; (b) Unnecessary operation especially in the female, J. Henry Dowd.

*Section on Surgery.*—Tuesday evening, March 5. Program: (a) A working formula for the treatment of intraperitoneal infection, William B. Jones, Attending Surgeon St. Mary's Hospital, Rochester, N. Y.; (b) Typhoid ulceration, John Parmenter.

